# Effects of Digital Technologies on Older People’s Access to Health and Social Care: Umbrella Review

**DOI:** 10.2196/25887

**Published:** 2021-11-24

**Authors:** Tafadzwa Patience Kunonga, Gemma Frances Spiers, Fiona R Beyer, Barbara Hanratty, Elisabeth Boulton, Alex Hall, Peter Bower, Chris Todd, Dawn Craig

**Affiliations:** 1 National Institute for Health Research Older People and Frailty Policy Research Unit Population Health Sciences Institute Newcastle University Newcastle upon Tyne United Kingdom; 2 National Institute for Health Research Older People and Frailty Policy Research Unit School of Health Sciences, Faculty of Biology, Medicine and Health The University of Manchester Manchester United Kingdom

**Keywords:** digital health, social care, access, older adults, review of reviews, umbrella review

## Abstract

**Background:**

The 2020 COVID-19 pandemic prompted the rapid implementation of new and existing digital technologies to facilitate access to health and care services during physical distancing. Older people may be disadvantaged in that regard if they are unable to use or have access to smartphones, tablets, computers, or other technologies.

**Objective:**

In this study, we synthesized evidence on the impact of digital technologies on older adults’ access to health and social services.

**Methods:**

We conducted an umbrella review of systematic reviews published from January 2000 to October 2019 using comprehensive searches of 6 databases. We looked for reviews in a population of adults aged ≥65 years in any setting, reporting outcomes related to the impact of technologies on access to health and social care services.

**Results:**

A total of 7 systematic reviews met the inclusion criteria, providing data from 77 randomized controlled trials and 50 observational studies. All of them synthesized findings from low-quality primary studies, 2 of which used robust review methods. Most of the reviews focused on digital technologies to facilitate remote delivery of care, including consultations and therapy. No studies examined technologies used for first contact access to care, such as online appointment scheduling. Overall, we found no reviews of technology to facilitate first contact access to health and social care such as online appointment booking systems for older populations.

**Conclusions:**

The impact of digital technologies on equitable access to services for older people is unclear. Research is urgently needed in order to understand the positive and negative consequences of digital technologies on health care access and to identify the groups most vulnerable to exclusion.

## Introduction

For at least a decade, the World Health Organization has encouraged member states to become leaders in serving citizens online, using digital technology to improve health and social care services [1]. Digital technologies are electronic tools, systems, and resources that generate, store, or process data [2]. The emergence of a novel coronavirus (SARS-CoV-2, which causes COVID-19) has led to the rapid rollout of digital technologies to support patient access to health and social care, while ensuring physical distancing [3]. Digital technologies were playing a growing role in connecting health and social care services with their users before the COVID-19 pandemic. A survey of patients aged 65 years and over in 9 countries in 2013 reported that over three-quarters preferred to book and manage their medical appointments online, and over three-quarters felt that online access to medical records was important [4]. The annual survey of 770,000 patients in UK family practice has described small increases in the proportion of people booking appointments (14.9% in 2019, up from 12.9% in 2018) and ordering repeat prescriptions online (16.2% in 2019, up from 14.3% in 2018) [5].

Supporting people to use digital health resources may help improve access to services, improve physical and mental well-being, and encourage shared decision-making [5]. However, estimates suggest that 37% of the world’s estimated 7.8 billion population are digitally excluded [6], with older people, people on low incomes, and other marginalized groups most likely to be affected [5,7]. In the United States, around 80% of the population accesses the internet, but its use falls sharply with increasing age. Approximately 70% of the people aged 65 to 74 years are online, compared with 52% of those aged 75 to 84 years, and 38% aged ≥85 years [8]. In the United Kingdom, out of a total population of 66.4 million, approximately 11 million (20%) lack digital skills, and 8.4 million (8.5%) never go online [9], and just over half of the latter are aged over 65 [5]. There is a clear relationship between internet use and health, with increasing age, female gender, and greater deprivation being associated with lower internet use [10]. Potential barriers to digital access include lack of awareness, confidence, capacity, or skills [11,12], a reluctance to change established behaviors, and poor internet access [5]. Affordability and acceptability of digital technology is important in later life, and it is noteworthy that many devices have been developed without the involvement of older people [13]. The involvement of older adults in technological design and development can facilitate acceptability, although it is a complex matter and requires careful consideration [14].The recent widespread introduction of digital alternatives to face-to-face interactions makes it vital that we understand their impact on older adults’ ability to access health and social care services that they need. In the United Kingdom, the National Health Service (NHS) roadmap sets out the milestones for digital health and social care to support people to live healthier lives and use fewer care services using technologies such as mobile phones and smartphones, tablets, and smart televisions [15]. It includes NHS digital health and wellbeing apps, such as the NHS app, which provides access to a range of NHS services via smartphones or tablets, and NHS login, which allows patients to view and access their personal health information online [16]. These technologies could potentially improve access to services by (1) facilitating first contact with services, (2) replacing face-to-face care with remote service delivery, and (3) providing access to professional support through remote patient monitoring [2]. Therefore, this review of reviews aims to answer the question of whether digital technologies improve access to health and social care for older adults and identify the characteristics of any digital interventions that are effective in increasing access to services for older adults.

## Methods

### Reporting Standards

We employed an umbrella review methodology to summarize the findings of previously published reviews [17]. The review adheres to the PRISMA (Preferred Reporting Items for Systematic Reviews and Meta-Analysis) checklist for the reporting of systematic reviews [18]. The PRISMA checklist for this study is provided in Multimedia Appendix 1. Moreover, a review protocol was registered in the PROSPERO database [19].

### Inclusion Criteria

The inclusion criteria were based on the PICOS (Population, Interventions, Comparator, Outcomes, and Study Designs) [20] criteria, which will be described in the following section.

#### Participants

Reviews of studies on older adults aged ≥65 or a combination of older and younger populations were selected in order to compare the effects of digital technologies on health care access between younger and older people.

#### Intervention

We used studies on any form of digital technology intended to facilitate access to appropriate health and social care services. These technologies enable first contact access (eg, online appointment scheduling) and are used as platforms for consultations and therapy interventions. They are also used in the remote care of patients. Furthermore, we recognized the fact that access to health and social care services would encompass availability and supply (ie, the degree of availability and quantity of supply at hand, regardless of whether they are used), utilization, equity, effectiveness, and quality of care [21].

#### Outcomes

We aimed to study the impact of digital technology on access to health and social care, which included the changes made in access and use of services as well as the cost-effectiveness of interventions that facilitate access and delivery of health and social care.

#### Study Designs

The study design of this paper encompassed any type of systematic review.

### Search Strategy

We searched the following databases: Epistemonikos, MEDLINE (Ovid), Cochrane Database of Systematic Reviews (Wiley), ASSIA (ProQuest), PROSPERO, and for gray literature in Health Management Information Consortium (Ovid) and King’s Fund. We used thesaurus headings along with title and abstract terms to search for digital technologies combined with specified outcomes for older people. The Canadian Agency for Drugs and Technologies in Health systematic review filter was adapted for databases that contained multiple study designs [22]. Searches were limited to the English language and the material published from January 1, 2000, to October 2019. The MEDLINE strategy is reported in Multimedia Appendix 2. The search results were downloaded to Endnote X9 (Clarivate Analytics) and deduplicated.

### Data Collection

Two-stage screening was conducted by 2 reviewers independently using the Rayyan (Rayyan Systems) systematic review application [23]. We first tested and refined the inclusion and exclusion criteria on a sample of titles and abstracts to ensure that they were robust enough to capture relevant articles. The titles and abstracts of the reviews were screened against the refined inclusion criteria, followed by full text assessment of the selected articles. We resolved disagreements between the reviewers by discussion or by arbitration from another member of the review team.

### Data Extraction

We extracted data into an Excel (Microsoft Corporation) spreadsheet, using a form based on the Cochrane Data Extraction and Assessment Template [20] to record the relevant review characteristics. The extracted data included: (1) author and year of publication; (2) title; (3) objective of the review; (4) description of the included population; (5) total number of older people; (6) intervention; (7) technology type; (8) what the intervention is enhancing; (9) primary outcomes; (10) secondary outcomes; (11) overall statement on quality appraisal; and (12) review authors’ summary. To ensure comprehensiveness, we piloted the abstraction form on 2 reviews, which identified a need for minor modifications. Risk of bias was assessed using the ROBIS (Risk of Bias in Systematic Reviews) tool [24]. We chose to use ROBIS as opposed to AMSTAR 2 (A Measurement Tool to Assess Systematic Reviews) because we are experienced with the former, and a comparative analysis of the two tools showed little difference between them [25].

### Data Analysis

We presented our main results in tabular format with a narrative synthesis. We grouped the results according to the three purposes of digital health technology, which consist of enabling first contact access, consultations and therapy, and remote monitoring. Due to a lack of data, we were unable to analyze the effects of interventions at ages over 65 years.

## Results

**Figure 1:**
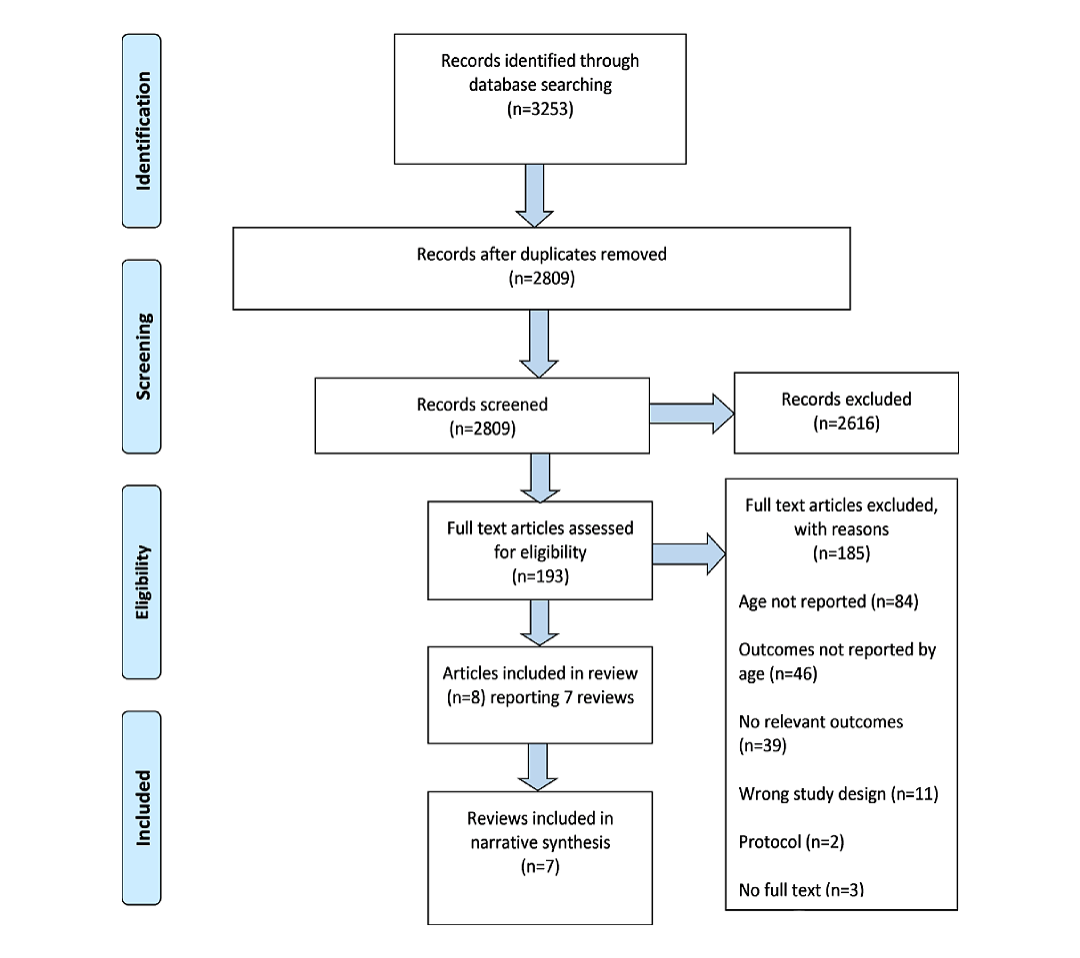
PRISMA (Preferred Reporting Items for Systematic Reviews and Meta-Analysis) flow diagram.

Database searches identified 2809 unique records. The initial screening of title and abstracts excluded 2616 records, leaving 193 for full text assessment (Figure 1). We identified 7 reviews eligible for inclusion.

### Characteristics of the Included Reviews

A total of 7 reviews published between 2006 and 2019 met the inclusion criteria [26-32]. A descriptive summary of review characteristics is presented in Table 1. The 7 included reviews include a total of 77 randomized controlled trials (RCTs) and 50 observational studies. We assessed the overlap across the reviews and identified 7 RCTs [33-39] reported in more than 1 review, but no observational studies that were included in more than 1 review. The studies in the reviews included 49 from the United States, 40 from Europe (including 7 from the United Kingdom), 9 from Australia, 6 from Canada, and 6 from the rest of the world. Country of origin was not stated for the remaining 17 studies. All of the studies reported outcomes for adults aged 65 and older, and 2 reviews included adults from age 18 [26,32].

**Table 1:** Summary of included systematic reviews.

Author	Study designs included in the review	Population	Intervention	Type of technology	Outcome
Bauce [26]	RCT,^a^ observational	Adults aged >65 (in 10 out of 11 studies)	Telemonitoring	Videophones, smartphone, and mobile phone	Hospital admissions and emergency department visits
Harerimana [27]	RCT, observational	Adults aged ≥65 with a diagnosis of depression or self-reported depressive symptoms	Telehealth (mental health)	Telephone and computers	Hospital admissions and emergency department visits
Husebo [28]	Observational	Adults aged >65, either living alone or receiving informal care	Telehealth	Videophones, personal computers or laptops, and TV	Hospital admissions and readmissions
Inglis [29,40]	RCT	Adults with heart failure (8 studies included people with a mean age of ≥70)	Structured telephone support or telemonitoring (heart failure)	Telephone	Heart failure and all-cause hospitalizations
Martinez [30]	RCT, observational	Adults with heart failure (11 studies included people with a mean age of ≥65)	Home telecare	Not reported	Hospital readmissions
Marx [31]	RCT, observational	Adults with a mean age of ≥65 years living independently, in receipt of intervention for management risk of malnutrition	Telehealth for managing risk of malnutrition	Telephone and computer	Hospital readmission and healthcare costs
Sanyal [32]	RCT, observational	Older adults (11 studies included people with a mean age of ≥65 years)	Telehealth, cognitive behavior therapy	Computer	Cost-effectiveness or utility of eHealth technologies

^a^RCT: randomized controlled trial.

### Risk of Bias Assessment

Details of the risk of bias assessment can be found in Table 2. Overall, the risk of bias was high for 5 reviews [26-28,30,32], and low for 2 reviews [29,31]. The main issues were the absence of clear inclusion criteria and the lack of publicly available protocols with predefined criteria. A detailed description of risk of bias assessment is reported in Multimedia Appendix 3.

**Table 2:** Risk of bias using ROBIS (Risk of Bias in Systematic Reviews) assessment.

Review	Phase 2					Phase 3
	Study Eligibility Criteria	Identification and selection of studies	Data collection and study appraisal	Synthesis and findings	Overall risk of bias	
Bauce [26]	High	Unclear	High	High	High	
Harerimana [27]	High	Unclear	High	High	High	
Husebo [28]	High	High	High	High	High	
Inglis [29,40]	Low	Low	Low	Low	Low	
Martinez [30]	High	High	High	High	High	
Marx [31]	Low	Low	Low	Low	Low	
Sanyal [32]	High	Low	Unclear	High	High	

### Outcomes

Table 3 summarizes the identified evidence, presenting it according to the purpose of the digital technology and the reported outcomes. None of the reviews reported outcomes that were related to the changes in access to services. In total, 6 reviews reported on hospital admissions [26-31], 1 reported on healthcare costs [31], and 1 on the cost-effectiveness of digital technology [32]. A variety of digital technologies were used by healthcare professionals and older adults to support interventions for telemonitoring or telecare: videophones or video conferencing equipment, internet-based applications, and smartphones.

**Table 3:** Overview of the identified evidence by type of digital technology and outcome.

Objective	Outcome	
Purpose of digital technology	Health service utilization	Costs and cost-effectiveness
Digital technology to enable first point of contact access (eg, online appointment scheduling)	No reviews identified	No reviews identified
Digital technologies or platforms for consultations and therapy interventions	Harerimana [27]; Marx [31]; Husebo [28]; Inglis [29]; Martinez [30]	Sanyal [32]
Digital technology for remote monitoring interventions	Bauce [26]	Sanyal [32]

#### First Point of Contact Access

No systematic reviews reported evidence about the impact of digital technology to facilitate first point of contact access with health services, such as online appointment scheduling.

#### Consultations and Therapies

In total, 5 reviews reported on health care service utilization, in malnutrition [31], heart failure [29,30], and mental health [27,28], as outcomes of digital technologies, but only 2 reviews were judged to be at low risk of bias and thus of higher quality [29,31].

#### Malnutrition

Marx and colleagues [31] reported weak evidence for the effectiveness of telehealth interventions to address malnutrition among community-dwelling older adults. They identified 9 studies (7 RCTs and 2 observational); 2 of the 9 studies reported significant reductions in hospital readmissions in the intervention groups. However, when the data were pooled, the reduction in hospital admissions was not significant; (odds ratio 0.52, 95% CI 0.24-1.16); *P*=.11; n=160; I^2^=0%).

#### Heart Failure

Inglis and colleagues [29] focused on whether structured telephone support and telemonitoring were effective for older people with heart failure. They found 41 RCTs that assessed heart failure–related hospitalizations. A meta-analysis of some of the included studies reported a 15% reduction in risk for heart failure–related hospitalizations with structured telephone support (relative risk 0.85, 95% CI 0.77-0.93; n=7030; 16 studies; I^2^=27%) and a 29% reduction in telemonitoring (relative risk 0.71, 95% CI 0.60 to 0.83; n=2148; 8 studies; I²=20%). There were no impacts reported on all-cause hospitalizations. The quality of the evidence reported for these heart failures and all-cause hospitalization studies was rated very low [29]. Evidence from the lower-quality reviews reported positive impacts of digital technology interventions on service utilization. Martinez and colleagues [30] reviewed 42 articles on the value of home monitoring for heart failure patients, 5 of which reported findings for older people. Remote consultations and follow-up care were associated with lower admission and readmission rates.

#### Mental Health

Husebo and colleagues [28] sought to understand the care content and utilization of virtual visits, particularly the uses and experiences of adults aged 65 and over. In their review, 1 study reported that all-cause readmissions were lower in the telehealth group (n=102) compared with standard care (n=116). At 30 days, 16 (16%) versus 22 (19%) and at over 6 months, 46 (46%) versus 60 (52%) of intervention versus control patients were readmitted [41]. Telehealth has also been used to deliver mental health care for older adults with depressive symptoms (telemental health). In a 6-month single (quasi-experimental) study of 76 patients, identified by Harerimana and colleagues [27], telemental health reduced hospital admissions by 80% (46 versus 9 admissions) and emergency room visits 60% (80 versus 32 visits) [42]. Evidence for the impact of digital technologies on economic outcomes was sparse. Moreover, 1 single review of eHealth technologies in the management of chronic diseases reported limited evidence, which did not support the assessment of cost-effectiveness [32].

#### Remote Monitoring

Two reviews reported evidence about technologies for remote monitoring, both of which were judged to be of poor quality. Bauce and colleagues [26] assessed the effectiveness of telemonitoring (videoconferencing) interventions on heart failure outcomes in 11 studies (10 RCTs and 1 single-group study). Five studies reported significant reductions in hospital admissions, and 2 others reported significant reductions in emergency department visits. The authors speculated that the reduction in healthcare use was likely to be due to the early detection and treatment of symptoms attributable to the intervention. Reduction in hospital admissions due to telemonitoring was supported by Queirós and colleagues [43]. Their systematic review assessed the use of technologies in the remote care of patients with long-term conditions such as diabetes, congestive heart failure, chronic obstructive pulmonary disease, and mental disorders [43].

## Discussion

### Principal Results

We identified evidence on a variety of digital technologies to facilitate interaction between older people and services at different parts of the care pathway. However, we found no reviews of technology to facilitate first point of contact access such as online appointment booking systems. There was no significant difference in hospital admissions for telehealth interventions (but this may have been due to the studies’ lack of power as there were only 160 participants in the pooled analysis) [31]. However, for heart failure, structured telephone support resulted in 15% reduction in admissions [29]. Other reviews were of too low a quality to permit confidence in findings, however, and there were no signs that a focus on reviews with too low a risk of bias would change anything. From the 7 overlapping RCTs [33-39], benefits to the older population in access were poorly measured and not clearly reported. In these RCTs, focus was on reducing hospital admissions, and there was little account of whether these technologies are enabling older people to interact with or access health and social care services more effectively. There was also no review evidence for newer technologies such as smartphone apps (eg, the NHS app in the United Kingdom), some of which were already in widespread use before COVID-19 [15].

The 2020 COVID-19 pandemic prompted the rapid implementation of alternatives to face-to-face interactions in health and social care [3]. This was a pragmatic response to a novel emergency that allowed care delivery to continue. As the pandemic evolves, digital innovations that have been implemented at speed should be evaluated to ensure that they are effective and affordable so that they can promote equitable access and do not selectively overlook certain sections of the population [14]. However, none of the included reviews addressed the issue of affordability and acceptability of digital technology in later life. For sections of the population who lack digital literacy or a means of digital engagement, the benefits are less clear, and there is every possibility that they will be harmed by losing the ability to access services in traditional, nondigital ways.

Most of the evidence [26-31] was concerned with digital technologies to facilitate remote delivery of care, including consultations and therapy, reflecting a research focus congruent with policy priorities [15]. However, these evaluations were more focused on reducing hospital utilization rather than enhancing access to services. Whether digital technologies do reduce hospital admissions and visits by facilitating timely access to appropriate alternative care is impossible to determine from the evidence presented. Evidence on the cost-effectiveness of digital health technologies was confined to 1 low-quality review, from which no clear conclusions can be drawn [32].

### Limitations

To the best of our knowledge, this is the first rapid synthesis of systematic reviews on digital technology aimed at enhancing access to health and social care services for older adults. We followed a rapid evidence synthesis approach, and our database searching, handling of data, and reporting adhered to published guidelines for undertaking a robust standard systematic review [18,44]. We restricted our searches to English language publications due to time constraints and acknowledge that this may have excluded relevant material. Two limitations of the material should be highlighted. First, most of the studies contained within our included reviews were randomized trials of effectiveness and cost-effectiveness. However, we found that the benefits to the older population in access are poorly measured and not clearly reported in studies of digital technology. Second, most of the reviews failed to adequately report their findings, and formal assessments of the methodological quality indicated a low-quality evidence base. This leads us to be cautious in our interpretation of the evidence and any conclusions drawn.

### Comparison With Prior Work

Our assessment of the dearth of evidence on first point of contact digital technology is supported by other works. A recent review of approaches to the evaluation of digital health interventions identified little evidence from randomized controlled trials and carried out measurement of service utilization in only a minority of the studies [45]. Our review suggests that digital health technologies may be associated with reductions in health service use. This is supported by multiple systematic reviews in younger populations of patients with long-term conditions [43]. There is a particular gap in the evaluation of any digital technologies used in social care.

### Implications for Policy, Research, and Practice

The COVID-19 pandemic has resulted in the rapid implementation of digital interventions to allow continued access to services when infection risk was high. This rapid rollout went beyond any evidence for effectiveness, driven by the extraordinary need to reduce face-to-face contact. However, prepandemic concerns about the adverse effects of digital technologies on access to services for older people remain valid. For older people who are digitally excluded, these digital interventions risk exacerbating any problems they already faced when trying to access health and social care services. This, in turn, has implications for workload in primary care, and health care providers must take on greater responsibility to ensure that this important section of the population receives the care it needs. There is a notable gap in the evidence for studies assessing the impact of technologies to enable first point of contact for health and social care services (eg, online platforms to book appointments). A mapping review of primary studies is required to understand this impact on different population subgroups, but this is unlikely to be sufficient. Further work is needed to understand the effectiveness and cost-effectiveness of digital technologies and their effect on equity of access to health and social care services. This should encompass access to appropriate care, which may lead to reductions in the use of other services as well as changes in health outcomes. The paucity of evidence in this area points to the need for a broad research program in partnership with older people and service providers in order to understand the characteristics of digital technologies, which can enhance access to services.

### Conclusions

The current systematic review evidence on the potential for digital technologies to improve access to health and social care for older adults is limited in both scope and quality. However, these limited attempts raise the possibility that providing digital interventions in addition to or as a replacement for face-to-face services may reduce demands on hospitals. Further research is required, and the widespread use of digital technologies to facilitate access to health and social care during the COVID-19 pandemic offers an ideal opportunity to better understand the barriers, facilitators, and limitations of their use.

## Appendix

Multimedia Appendix 1 PRISMA (Preferred Reporting Items for Systematic Reviews and Meta-Analysis) checklist.

Multimedia Appendix 2 Example of search strategy.

Multimedia Appendix 3 Risk of bias assessment of the included reviews.

## Abbreviations

AMSTAR: A Measurement Tool to Assess Systematic Reviews
NHS: National Health Service
NIHR: National Institute for Health Research
PICOS: Population, Interventions, Comparator, Outcomes, and Study Designs
PRISMA: Preferred Reporting Items for Systematic Reviews and Meta-Analysis
RCT: randomized control trial
ROBIS: Risk of Bias in Systematic Reviews
