# Supplementary material for: Effects of Digital Technologies on Older People’s Access to Health and Social Care: Umbrella Review
Source: J Med Internet Res. 2021 Nov 24;23(11):e25887. doi: 10.2196/25887 (PMC8663708; doi:10.2196/25887)
Supplement: Multimedia Appendix 2 [file jmir_v23i11e25887_app2.docx]

# Multimedia Appendix 1

MEDLINE search strategy

Database(s): Ovid MEDLINE(R) and In-Process & Other Non-Indexed Citations 1946 to October 17, 2019
Searched 18^th^ October 2019

| **#** | **Searches** | **Results** |
| --- | --- | --- |
| 1 | Internet/ | 70118 |
| 2 | Social Media/ | 6511 |
| 3 | Mobile Applications/ | 4654 |
| 4 | Wearable Electronic Devices/ | 1422 |
| 5 | Computers/ | 50449 |
| 6 | exp Microcomputers/ | 20907 |
| 7 | Minicomputers/ | 979 |
| 8 | Therapy, Computer-Assisted/ | 6590 |
| 9 | Computer-Assisted Instruction/ | 11599 |
| 10 | exp Cell Phone/ | 9827 |
| 11 | Electronic Mail/ | 2592 |
| 12 | (e-BI or e-SBI or ehealth or e-health or electronic health or mhealth or m-health or mobile health or virtual health or digital health or digital technolog* or technological aid? or wearable*).ti,ab. | 32276 |
| 13 | ((email* or e-mail* or electronic mail* or text messag* or SMS or MMS or phone? or cellphone? or cell-phone? or smartphone? or smart-phone? or digital tablet? or pda or personal digital assistant? or social media or social networking or facebook or twitter or skyp* or app?) adj2 (deliver* or generat* or based or provid* or facilitat* or support* or treatment? or therap* or intervention? or program* or feedback)).ti,ab. | 11486 |
| 14 | ((Internet* or electronic* or digital* or online or on-line or computer* or laptop? or software or web*) adj2 (deliver* or generat* or based or provid* or facilitat* or support* or treatment? or therap* or intervention? or program* or feedback)).ti,ab. | 116926 |
| 15 | exp Telemedicine/ | 26136 |
| 16 | (telecare or tele care or telehealth or tele health or telemedicine or tele medicine or teleconsultation* or tele-consultation* or tele monitor* or telemonitor*).ti,ab,kw. | 15975 |
| 17 | or/1-16 | 306305 |
| 18 | Cost-Benefit Analysis/ | 78206 |
| 19 | exp "Patient Acceptance of Health Care"/ | 144454 |
| 20 | exp Health Services Accessibility/ | 106406 |
| 21 | Electronic Prescribing/ | 976 |
| 22 | exp "Appointments and Schedules"/ | 19476 |
| 23 | Healthcare Disparities/ | 15183 |
| 24 | Digital Divide/ | 42 |
| 25 | ((access* or utiliz* or utilis* or accept*) adj3 (health care or healthcare or primary care or secondary care or social care or community or service? or hospital* or clinic? or appointment* or referral* or consultation? or prescribing or prescription*)).ti,ab. | 74717 |
| 26 | or/18-25 | 394871 |
| 27 | 17 and 26 | 18279 |
| 28 | (digital* adj3 (inclusion or exclusion or divide or monitor* or deliver*)).ti,ab. | 1396 |
| 29 | ((digital* or online or internet* or computeri*) adj3 (access or engag* or disengag* or healthcare or health care or social care or service? or hospital* or clinic? or appointment* or referral* or consultation? or prescribing or prescription*)).ti,ab. | 10587 |
| 30 | (remote* adj3 (access or monitor*)).ti,ab. | 3772 |
| 31 | or/28-30 | 15476 |
| 32 | 27 or 31 | 32344 |
| 33 | exp Aged/ | 3002833 |
| 34 | Health Services for the Aged/ | 17328 |
| 35 | or/33-34 | 3004177 |
| 36 | 17 and 35 | 28230 |
| 37 | 32 or 36 | 56106 |
| 38 | meta-analysis.pt. | 106529 |
| 39 | meta-analysis/ or systematic review/ or meta-analysis as topic/ or "meta analysis (topic)"/ or "systematic review (topic)"/ or exp technology assessment, biomedical/ | 199881 |
| 40 | ((systematic* adj3 (review* or overview* or analys*)) or (methodologic* adj3 (review* or overview* or analys*))).ti,ab,kf,kw. | 177440 |
| 41 | (((quantitative or narrative*) adj3 (review* or overview* or synthes*)) or (research adj3 (integrati* or overview*))).ti,ab,kf,kw. | 22041 |
| 42 | ((integrative adj3 (review* or overview*)) or (collaborative adj3 (review* or overview*)) or (pool* adj3 analy*)).ti,ab,kf,kw. | 23610 |
| 43 | (data synthes* or data extraction* or data abstraction*).ti,ab,kf,kw. | 23918 |
| 44 | (handsearch* or hand search*).ti,ab,kf,kw. | 8743 |
| 45 | (mantel haenszel or peto or der simonian or dersimonian or fixed effect* or latin square*).ti,ab,kf,kw. | 24102 |
| 46 | (met analy* or metanaly* or technology assessment* or HTA or HTAs or technology overview* or technology appraisal*).ti,ab,kf,kw. | 8475 |
| 47 | (meta regression* or metaregression*).ti,ab,kf,kw. | 7596 |
| 48 | (meta-analy* or metaanaly* or systematic review* or biomedical technology assessment* or bio-medical technology assessment*).mp,hw. | 269525 |
| 49 | (medline or cochrane or pubmed or medlars or embase or cinahl).ti,ab,hw. | 194565 |
| 50 | (cochrane or (health adj2 technology assessment) or evidence report).jw. | 19576 |
| 51 | (comparative adj3 (efficacy or effectiveness)).ti,ab,kf,kw. | 12073 |
| 52 | (outcomes research or relative effectiveness).ti,ab,kf,kw. | 8603 |
| 53 | ((indirect or indirect treatment or mixed-treatment) adj comparison*).ti,ab,kf,kw. | 1941 |
| 54 | ((meta-narrative or meta-ethnograph* or mixed method* or critical or thematic or realist or framework) adj3 (review* or synthes*)).ti,ab,kf,kw. | 24483 |
| 55 | or/38-54 | 455644 |
| 56 | 37 and 55 | 2895 |
| 57 | limit 56 to (english language and humans and yr="2000 -Current") | 2293 |
