# Supplementary material for: Effects of Digital Technologies on Older People’s Access to Health and Social Care: Umbrella Review
Source: J Med Internet Res. 2021 Nov 24;23(11):e25887. doi: 10.2196/25887 (PMC8663708; doi:10.2196/25887)
Supplement: Multimedia Appendix 3 [file jmir_v23i11e25887_app3.docx]

# Multimedia Appendix 2

Risk of Bias Assessment of Included Reviews

Domain 1: Study eligibility criteria

We assessed concerns regarding specification of the study eligibility criteria and five reviews were judged to be of high risk [26-28,30,32]. The main issues were the absence of clear inclusion criteria, and/or the lack of publicly available protocols with predefined criteria. Protocols help to reduce bias in the conduct of the review and promote consistency and transparency.

Domain 2: Identification and selection of studies

We assessed concerns regarding methods used to identify and select studies and two of the reviews in this domain were judged to be high risk [28,30], two as unclear [26,27] and three as low risk [29,31,32]. The main concerns in this domain were the restriction to English language studies [28], a lack of information about study selection [30], failure to specify date ranges for study searches [26], and insufficient detail on the numbers of reviewers involved in study selection [27]. Unbiased selection criteria would ensure that all relevant studies are included in the reviews, regardless of language or age of study. Ideally, study selection would include independent assessment of the studies for inclusion by at least two reviewers. Not only does this minimise bias, but it also ensures that decisions are checked for accuracy.

Domain 3: Data collection and study appraisal

We assessed concerns regarding methods used to collect data and appraise studies and, we rated four reviews as being at high risk of bias [26-28,30], one as unclear [32], and one as low risk [29]. The main problem within this domain was the failure to specify whether data collection and quality assessment were undertaken independently by more than one person. An unbiased data extraction process would ensure that mistakes are identified and there is no deviation from an agreed extraction form. As with data extraction, it is important for at least two reviewers to carry out risk of bias assessments to minimise errors.

Domain 4: Synthesis and findings

We assessed concerns regarding the synthesis and findings, and five of the reviews were rated as high risk of bias [26-28,30], and two as low risk [29,31]. The absence of clear outcome criteria and publicly available protocols made it difficult to be confident that the synthesis and reporting of outcomes was unbiased. Bias may be introduced if reviewers decide to synthesise outcomes based on their interpretation of the data. A pre-defined approach to analysis and synthesis ensures that bias is minimised.
